# Complete genome sequence of *Halorhodospira halophila* SL1

**DOI:** 10.4056/sigs.3677284

**Published:** 2013-04-15

**Authors:** Jean F. Challacombe, Sophia Majid, Ratnakar Deole, Thomas S. Brettin, David Bruce, Susana F. Delano, John C. Detter, Cheryl D. Gleasner, Cliff S. Han, Monica Misra, Krista G. Reitenga, Natalia Mikhailova, Tanja Woyke, Sam Pitluck, Matt Nolan, Miriam L. Land, Elizabeth Saunders, Roxanne Tapia, Alla Lapidus, Natalia Ivanova, Wouter D. Hoff

**Affiliations:** 1 Los Alamos National Laboratory and DOE Joint Genome Institute, Bioscience Division, Los Alamos, New Mexico, USA; 2 Department of Biochemistry and Molecular Biology, the University of Chicago, Chicago Illinois, USA; 3 Department of Microbiology and Molecular Genetics, Oklahoma State University, Stillwater, Oklahoma, USA; 4 DOE Joint Genome Institute, Walnut Creek, California, USA; 5 Oak Ridge National Laboratory, Oak Ridge, Tennessee, USA; 6 Lawrence Berkeley National Laboratory, Berkeley, CA, USA; 7 Argonne National Laboratory, Argonne, IL, USA.; 8 Noblis, National Security and Intelligence, Falls Church, Virginia, USA; 9 Fox Chase Cancer Center, Philadelphia, Pennsylvania, USA; 10 Northeastern State University, Broken Arrow, Oklahoma, USA

**Keywords:** halophile, saturated salt, sulfur metabolism, purple sulfur bacterium, phototrophic

## Abstract

*Halorhodospira halophila* is among the most halophilic organisms known. It is an obligately photosynthetic and anaerobic purple sulfur bacterium that exhibits autotrophic growth up to saturated NaCl concentrations. The type strain *H. halophila* SL1 was isolated from a hypersaline lake in Oregon. Here we report the determination of its entire genome in a single contig. This is the first genome of a phototrophic extreme halophile. The genome consists of 2,678,452 bp, encoding 2,493 predicted genes as determined by automated genome annotation. Of the 2,407 predicted proteins, 1,905 were assigned to a putative function. Future detailed analysis of this genome promises to yield insights into the halophilic adaptations of this organism, its ability for photoautotrophic growth under extreme conditions, and its characteristic sulfur metabolism.

## Introduction

*Halorhodospira halophila* is an anoxygenic photosynthetic halophile that was isolated from salt-encrusted mud along the shore of Summer Lake in Oregon [1], and from the hypersaline Wadi Natrun lakes in Egypt [2]. The original name of this organism, *Ectothiorhodospira halophila,* was modified to *Halorhodospira halophila* when the genus *Ectothiorhodospira* was divided into two genera (*Ectothiorhodospira* and *Halorhodospira*), and *E. halophila* was reclassified as a member of the genus *Halorhodospira*, serving as the type species of the new genus [3]. Over the last decade, the genomes of a number of extremely halophilic *Archaea* have been sequenced and analyzed, including *Halobacterium salinarum* [4,5], *Haloarcula marismortui* [6], *Natronomonas pharaonis* [7], and *Haloquadratum walsbyi* [8]. In addition, the genomes of three halophilic *Bacteria* have become available: *Salinibacter ruber* [9], *Halothermothrix orenii* [10], and ‘*Halanaerobium hydrogenoformans’* [11]. All of these organisms are obligate chemotrophs. Thus, *H. halophila* is the first phototrophic extreme halophile to have its genome sequence determined and analyzed. In contrast to other extreme halophiles that grow well in saturated salt concentrations, *H. halophila* has a high flexibility with respect to the salt concentrations that it tolerates, and grows optimally at all NaCl concentrations from 15% to 35%, with growth down to 3.5% NaCl [12]. In contrast, the above extremely halophilic archaea and *S. ruber* require 15% NaCl for growth.

*H. halophila* is of significant interest because it is an obligately anaerobic purple sulfur bacterium, and among the most halophilic organisms known [13]. To date, genome sequences are available for two phototrophic purple sulfur bacteria, *Allochromatium vinosum* DSM 180 and the *H. halophila* SL1 genome reported here. *H. halophila* has very few growth requirements. However, it does need reduced sulfur compounds for growth, as does *A. vinosum* [14]. Its pathways for both photosynthetic electron transfer [15-17] and nitrogen fixation [18] have attracted attention. In addition, *H. halophila* contains photoactive yellow protein [19,20]. This is the first member of a novel class of blue light receptors, and triggers a negative phototaxis response in *H. halophila* [21]. The photoactive yellow protein (PYP) from *H. halophila* has been studied extensively for its biophysical characteristics [22-24].

The sulfur metabolism of *H. halophila* is unusual, resulting in the transient accumulation of extracellular sulfur globules via metabolic pathways that are not yet fully resolved [14]. While purple non-sulfur phototrophs such as *Rhodobacter sphaeroides* and *Rhodospirillum rubrum* use organic compounds like malate as electron donors, *H. halophila* obtains electrons from reduced sulfur compounds. The genome sequence of *H. halophila* promises to reveal insights into its adaptations to hypersaline environments, and to allow a better understanding of its unique combination of metabolic capabilities, combining properties from extreme halophiles, anoxygenic phototrophs, and purple sulfur bacteria.

## Classification and features

**Figure 1:**
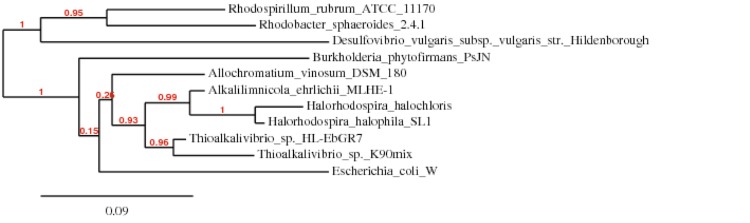
Phylogram representation of a phylogenetic tree highlighting the position of *Halorhodospira halophila* strain SL1 relative to other organisms of interest, including members of the *Ectothiorhodospiraceae*, as well as additional strains that were included for comparison purposes, based on environmental and functional considerations. The strains (type=^T^) and their corresponding GenBank accession numbers (and coordinates) for 16S rRNA genes are: *H. halophila* strain SL1^T^, CP00544:380025-381562; *Alkalilimnicola ehrlichii* strain MLHE-1, CP00453:369818-369894; *Thioalkalivibrio sp.* HL-EbGR7, CP001339:2548250-2549775; *Thioalkalivibrio sp.* K90mix, CP001905:423231-424758; *Allochromatium vinosum* DSM 180^T^, CP001896:112452-113967; *Ectothiorhodospira halochloris* M59152; *Burkholderia phytofirmans* PsJN, CP001052:1541578-1543101; *Desulfovibrio vulgaris subsp. vulgaris* strain Hildenborough, AE017285:105921-107426; *Rhodobacter sphaeroides* 2.4.1, CP000143:1-1464; *Rhodospirillum rubrum* ATCC 11170, CP000230: 192528-194004; *Escherichia coli* B strain REL606, CP000819: 226609-228150. The 16S rRNA sequences were aligned by MUSCLE [37]. The tree was determined by the maximum likelihood model of PhyML [38] and rendered with TreeDyn [39], using the “one click” pipeline of the Phylogeny.fr web resource [40].

*H. halophila* belongs to the *Gammaproteobacteria* [3] (Table 1). The 16S rRNA gene sequence of *H. halophila* SL1 reveals closer relationships with *H. halochloris* and *Alkalilimnicola ehrlichii,* the other representatives of the *Ectothiorhodospiraceae* (Figure 1), than with *A. vinosum,* a purple sulfur bacterium in the *Chromatiaceae* family, and the haloalkaliphilic chemolithoautotrophic *Thioalkalivibrio* strains.

**Table 1:** Classification and general features of *H. halophila* SL1 according to the MIGS recommendations [25].

**MIGS ID**	**Property**	**Term**	**Evidence code**^a^
	Current classification	Domain *Bacteria*	TAS [26]
		Phylum *Proteobacteria*	TAS [27]
		Class *Gammaproteobacteria*	TAS [28,29]
		Order *Chromatiales*	TAS [28,30]
		Family *Ectothiorhodospiraceae*	TAS [31]
		Genus *Halorhodospira*	TAS [32-34]
		Species *Halorhodospira halophila*	TAS [32,33]
MIGS-7	Subspecific genetic lineage	DSM 244^T^	
	Gram stain	negative	NAS
	Cell shape	spiral	TAS [1]
	Motility	motile	TAS [1]
	Sporulation	non-sporulating	NAS
	Temperature range	mesophilic	NAS
	Optimum temperature	47°C	TAS [1]
	Carbon source	CO_2_, succinate, acetate	TAS [35]
	Energy source	photosynthesis	TAS [1]
MIGS-6	Habitat	salt lake mud	TAS [1]
MIGS-6.3	Salinity	Extreme halophile	TAS [1]
MIGS-22	Oxygen	anaerobe	TAS [1]
MIGS-15	Biotic relationship	free living	NAS
MIGS-14	Pathogenicity	none	NAS
MIGS-4	Geographic location	Summer Lake, Lake County, OR	TAS [1]
MIGS-5	Sample collection time	about 1967	TAS [1]
MIGS-4.1	Latitude	not reported	
MIGS-4.2	Longitude	not reported	
MIGS-4.3	Depth	not reported	
MIGS-4.4	Altitude	not reported	

^a^Evidence codes - IDA: Inferred from Direct Assay; TAS: Traceable Author Statement (i.e., a direct report exists in the literature); NAS: Non-traceable Author Statement (i.e., not directly observed for the living, isolated sample, but based on a generally accepted property for the species, or anecdotal evidence). These evidence codes are from the Gene Ontology project [36]. If the evidence code is IDA, then the property should have been directly observed, for the purpose of this specific publication, for a live isolate by one of the authors, or an expert or reputable institution mentioned in the acknowledgements.

## Genome sequencing and annotation

### Genome project history

This organism was selected for sequencing to better understand its halophilic adaptations, its unusual sulfur metabolism, its photosynthetic pathways, and to provide a framework for better understanding signaling pathways for photoactive yellow protein. The complete genome sequence has been deposited in GenBank. Sequencing, finishing and annotation were performed by the DOE Joint Genome Institute (JGI). Table 2 presents the project information and its association with MIGS version 2.0 compliance [25].

**Table 2:** Project information

**MIGS ID**	**Property**	**Term**
MIGS-31	Finishing quality	Finished
MIGS-28	Libraries used	40kb, 8kb, 3kb
MIGS-29	Sequencing platforms	Sanger
MIGS-31.2	Fold coverage	12×
MIGS-30	Assemblers	phrap
MIGS-32	Gene calling method	Critica
	Genbank ID	CP000544
	Genbank Date of Release	January 12, 2012
	GOLD ID	Gc00492
	Project relevance	extremophile

### Growth conditions and DNA isolation

*H. halophila* SL1 strain DSM 44^T^ was obtained from Deutsche Sammlung vor Mikroorganismen und Zellkulturen (DSMZ), Braunschweig, Germany, and were grown in DSMZ 253 medium. The cells were grown anaerobically and photosynthetically by placing them in 20 ml glass culture tubes completely filled with growth medium and sealed with screw caps. The tubes were kept at 42ºC in a water bath and illuminated with 70 W tungsten light bulbs. Chromosomal DNA was purified from the resulting cell cultures using the CTAB procedure.

### Genome sequencing and assembly

The random shotgun method was used in sequencing the genome of *H. halophila* SL1. Large (40 kb), median (8 kb) and small (3 kb) insert random sequencing libraries were sequenced for this genome project with an average success rate of 88% and average high-quality read lengths of 750 nucleotides. After the shotgun stage, reads were assembled with parallel phrap (High Performance Software, LLC). Possible mis-assemblies were corrected with Dupfinisher (unpublished, C. Han) or by transposon bombing of bridging clones (EZ-Tn5 <P6Kyori/KAN-2> Tnp Transposome kit, Epicentre Biotechnologies). Gaps between contigs were closed by editing, custom primer walks or PCR amplification. The completed genome sequence of *H. halophila* SL1 contains 36,035 reads, achieving an average of 12-fold sequence coverage per base with error rate less than 1 in 100,000.

### Genome annotation

Identification of putative protein-encoding genes and initial automated annotation of the genome was performed by the Oak Ridge National Laboratory genome annotation pipeline. Additional gene prediction analysis and functional annotation was performed within the IMG platform [41].

## Genome properties

**Figure 2:**
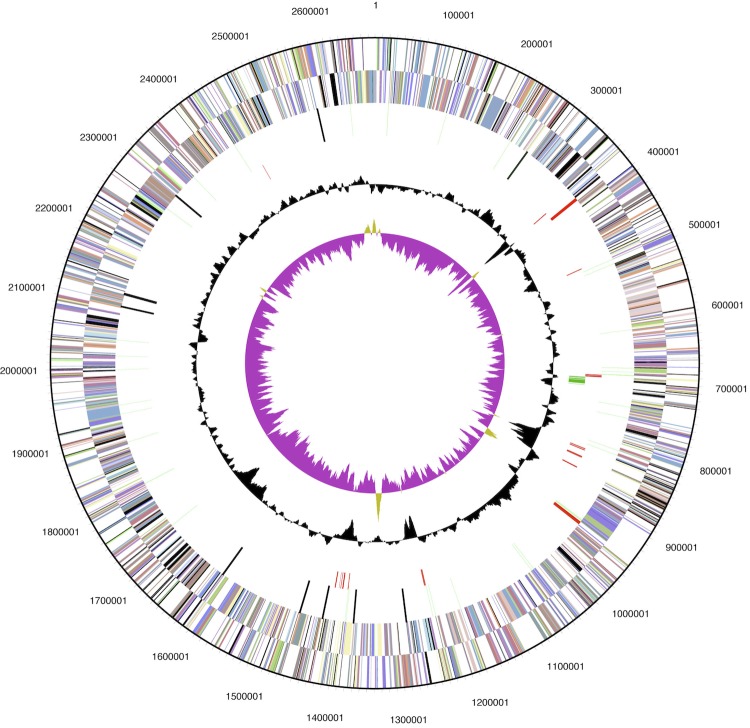
Graphical circular map of the genome. From outside to the center: Circle 1, genes on forward strand (colored by COG categories); Circle 2, genes on reverse strand (colored by COG categories); Circle 3, RNA genes (tRNAs green, rRNAs red, other RNAs black); Circle 4, mobile element genes; Circle 5, CRISPR-associated protein genes; Circle 6, GC content; Circle 7, GC skew.

The genome is 2,678,452 bp long and comprises one circular chromosome with 67% GC content (Figure 2). For the main chromosome, 2,493 genes were predicted, 2,407 of which are protein-coding genes. A total of 1,905 of protein coding genes were assigned to a putative function, with the remaining annotated as hypothetical proteins. In addition, 31 pseudo genes were identified. The properties and the statistics of the genome are summarized in Tables 3-4.

**Table 3:** Nucleotide content and gene count levels of the genome

**Attribute**	**Value**	**% of total**
Genome size (bp)	2,678,452	100.00%
DNA coding region (bp)	2,437,391	91%
DNA G+C content (bp)	1,794,562	67%
Total genes	2493	
RNA genes	63	2.65%
rRNA operons	2	
Protein-coding genes	2,407	96.55%
Pseudo genes	31	1.24%
Genes in paralog clusters	204	8.19%
Genes assigned to COGs	1,457	58.44%
Genes with signal peptides	499	20.02%
Genes with transmembrane helices	554	22.22%

**Table 4:** Number of genes associated with the 25 general COG functional categories

**Code**	**Value**	**%age**^a^	**Description**
J	147	5.9	Translation
A	1	0.0	RNA processing and modification
K	86	3.5	Transcription
L	125	5.0	Replication, recombination and repair
B	1	0.0	Chromatin structure and dynamics
D	36	1.4	Cell cycle control, mitosis and meiosis
Y	0	0.0	Nuclear structure
V	29	1.2	Defense mechanisms
T	156	6.3	Signal transduction mechanisms
M	144	5.8	Cell wall/membrane biogenesis
N	93	3.7	Cell motility
Z	0	0.0	Cytoskeleton
W	0	0.0	Extracellular structures
U	80	3.2	Intracellular trafficking and secretion
O	103	4.1	Posttranslational modification, protein turnover, chaperones
C	168	6.7	Energy production and conversion
G	76	3.1	Carbohydrate transport and metabolism
E	158	6.3	Amino acid transport and metabolism
F	46	1.9	Nucleotide transport and metabolism
H	152	6.1	Coenzyme transport and metabolism
I	72	2.9	Lipid transport and metabolism
P	122	4.9	Inorganic ion transport and metabolism
Q	37	1.5	Secondary metabolites biosynthesis, transport and catabolism
R	222	8.9	General function prediction only
S	167	6.7	Function unknown
-	493	19.8	Not in COGs

a The total is based on the total number of protein coding genes in the annotated genome.

## Conclusion

*H. halophila* is among the most halophilic eubacteria known. Further analysis and characterization of its genome will provide insights into the mechanisms it uses to adapt to hypersaline environments.

## References

[1] Raymond JC Sistrom WR The isolation and preliminary characterization of a halophilic photosynthetic bacterium. Arch Mikrobiol 1967; 59:255-268 10.1007/BF00406339 4880241

[2] Imhoff JF Hashwa F Trüper HG Isolation of extremely halophilic phototrophic bacteria from the alkaline Wadi Natrun, Egypt. Arch Hydrobiol 1978; 84:381-388

[3] Imhoff JF Suling J The phylogenetic relationship among *Ectothiorhodospiraceae*: A reevaluation of their taxonomy on the basis of 16S rDNA analyses. Arch Microbiol 1996; 165:106-113 10.1007/s002030050304 8593098

[4] Ng WV Kennedy SP Mahairas GG Berquist B Pan M Shukla HD Lasky SR Baliga NS Thorsson V Sbrogna J Genome sequence of *Halobacterium* species NRC-1. Proc Natl Acad Sci USA 2000; 97:12176-12181 10.1073/pnas.190337797 11016950 PMC17314

[5] Pfeiffer F Schuster SC Broicher A Falb M Palm P Rodewald K Ruepp A Soppa J Tittor J Oesterhelt D Evolution in the laboratory: the genome of *Halobacterium salinarum* strain R1 compared to that of strain NRC-1. Genomics 2008; 91:335-346 10.1016/j.ygeno.2008.01.001 18313895

[6] Baliga NS Bonneau R Facciotti MT Pan M Glusman G Deutsch EW Shannon P Chiu Y Weng RS Gan RR Genome sequence of *Haloarcula marismortui*: a halophilic archaeon from the Dead Sea. Genome Res 2004; 14:2221-2234 10.1101/gr.2700304 15520287 PMC525680

[7] Falb M Pfeiffer F Palm P Rodewald K Hickmann V Tittor J Oesterhelt D Living with two extremes: conclusions from the genome sequence of *Natronomonas pharaonis.* Genome Res 2005; 15:1336-1343 10.1101/gr.3952905 16169924 PMC1240075

[8] Bolhuis H Palm P Wende A Falb M Rampp M Rodriguez-Valera F Pfeiffer F Oesterhelt D The genome of the square archaeon *Haloquadratum walsbyi*: life at the limits of water activity. BMC Genomics 2006; 7:169 10.1186/1471-2164-7-169 16820047 PMC1544339

[9] Mongodin EF Nelson KE Daugherty S Deboy RT Wister J Khouri H Weidman J Walsh DA Papke RT Sanchez Perez G The genome of *Salinibacter ruber*: convergence and gene exchange among hyperhalophilic bacteria and archaea. Proc Natl Acad Sci USA 2005; 102:18147-18152 10.1073/pnas.0509073102 16330755 PMC1312414

[10] Mavromatis K Ivanova N Anderson I Lykidis A Hooper SD Sun H Kunin V Lapidus A Hugenholtz P Patel B Genome analysis of the anaerobic thermohalophilic bacterium *Halothermothrix orenii.* PLoS ONE 2009; 4:e4192 10.1371/journal.pone.0004192 19145256 PMC2626281

[11] Brown SD Begemann MB Mormile MR Wall JD Han CS Goodwin LA Pitluck S Land ML Hauser LJ Elias DA Complete genome sequence of the haloalkaliphilic, hydrogen-producing bacterium *Halanaerobium hydrogeniformans.* J Bacteriol 2011; 193:3682-3683 10.1128/JB.05209-11 21602336 PMC3133330

[12] Deole R, Challacombe J, Raiford DW, Hoff WD. An Extremely Halophilic Proteobacterium Combines a Highly Acidic Proteome with a Low Cytoplasmic Potassium Content. J Biol Chem 2012. 10.1074/jbc.M112.420505 PMC3537055 23144460

[13] Ollivier B Caumette P Garcia JL Mah RA Anaerobic bacteria from hypersaline environments. Microbiol Rev 1994; 58:27-38 8177169 10.1128/mr.58.1.27-38.1994 PMC372951

[14] Frigaard NU Dahl C Sulfur metabolism in phototrophic sulfur bacteria. Adv Microb Physiol 2009; 54:103-200 10.1016/S0065-2911(08)00002-7 18929068

[15] Leguijt T Engels PW Crielaard W Albracht SPJ Hellingwerf KJ Abundance, subunit composition, redox properties, and catalytic activity of the cytochrome bc1 complex from alkaliphilic and halophilic, photosynthetic members of the family *Ectothiorhodospiraceae.* J Bacteriol 1993; 175:1629-1636 8383662 10.1128/jb.175.6.1629-1636.1993 PMC203956

[16] Leguijt T Hellingwerf KJ Characterization of reaction center antenna complexes from bacteriochlorophyll a containing *Ectothiorhodospiraceae.* Biochim Biophys Acta 1991; 1057:353-360 10.1016/S0005-2728(05)80147-1

[17] Lieutaud C Alric J Bauzan M Nitschke W Schoepp-Cothenet B Study of the high-potential iron sulfur protein in *Halorhodospira halophila* confirms that it is distinct from cytochrome c as electron carrier. Proc Natl Acad Sci USA 2005; 102:3260-3265 10.1073/pnas.0407768102 15728382 PMC552902

[18] Tsuihiji H Yamazaki Y Kamikubo H Imamoto Y Kataoka M Cloning and characterization of nif structural and regulatory genes in the purple sulfur bacterium, *Halorhodospira halophila.* J Biosci Bioeng 2006; 101:263-270 10.1263/jbb.101.263 16716929

[19] Meyer TE Isolation and characterization of soluble cytochromes, ferredoxins and other chromophoric proteins from the halophilic phototrophic bacterium *Ectothiorhodospira halophila.* Biochim Biophys Acta 1985; 806:175-183 10.1016/0005-2728(85)90094-5 2981543

[20] Meyer TE Yakali E Cusanovich MA Tollin G Properties of a water-soluble, yellow protein isolated from a halophilic phototrophic bacterium that has photochemical activity analogous to sensory rhodopsin. Biochemistry 1987; 26:418-423 10.1021/bi00376a012 3828315

[21] Sprenger WW Hoff WD Armitage JP Hellingwerf KJ The Eubacterium *Ectothiorhodospira halophila* is negatively phototactic, with a wavelength dependence that fits the absorption spectrum of the photoactive yellow protein. J Bacteriol 1993; 175:3096-3104 8491725 10.1128/jb.175.10.3096-3104.1993 PMC204631

[22] Cusanovich MA Meyer TE Photoactive yellow protein: A prototypic PAS domain sensory protein and development of a common signaling mechanism. Biochemistry 2003; 42:4759-4770 10.1021/bi020690e 12718516

[23] Hellingwerf KJ Hendriks J Gensch T Photoactive Yellow Protein, a new type of photoreceptor protein: Will this "yellow lab" bring us where we want to go? J Phys Chem A 2003; 107:1082-1094 10.1021/jp027005y

[24] Kumauchi M Hara M Stalcup P Xie A Hoff WD Identification of six new photoactive yellow proteins: diversity and structure-function relationships in a bacterial blue light photoreceptor. Photochem Photobiol 2008; 84:956-969 10.1111/j.1751-1097.2008.00335.x 18399917

[25] Field D Garrity G Gray T Morrison N Selengut J Sterk P Tatusova T Thomson N Allen MJ Angiuoli SV The minimum information about a genome sequence (MIGS) specification. Nat Biotechnol 2008; 26:541-547 10.1038/nbt1360 18464787 PMC2409278

[26] Woese CR Kandler O Wheelis ML Towards a natural system of organisms: proposal for the domains *Archaea, Bacteria*, and *Eucarya.* Proc Natl Acad Sci USA 1990; 87:4576-4579 10.1073/pnas.87.12.4576 2112744 PMC54159

[27] Garrity GM, Bell JA, Lilburn T. Phylum XIV. *Proteobacteria* phyl. nov. In: Garrity GM, Brenner DJ, Krieg NR, Staley JT (eds), Bergey's Manual of Systematic Bacteriology, Second Edition, Volume 2, Part B, Springer, New York, 2005, p. 1.

[28] Validation of publication of new names and new combinations previously effectively published outside the IJSEM. List no. 106. Int J Syst Evol Microbiol 2005; 55:2235-2238 10.1099/ijs.0.64108-0 15879221

[29] Garrity GM, Bell JA, Lilburn T. Class III. *Gammaproteobacteria* class. nov. In: Garrity GM, Brenner DJ, Krieg NR, Staley JT (eds), Bergey's Manual of Systematic Bacteriology, Second Edition, Volume 2, Part B, Springer, New York, 2005, p. 1.

[30] Imhoff J. Order I. *Chromatiales* ord. nov. In: Garrity GM, Brenner DJ, Krieg NR, Staley JT (eds), Bergey's Manual of Systematic Bacteriology, Second Edition, Volume 2, Part B, Springer, New York, 2005, p. 1-3.

[31] Imhoff JF Reassignment of the Genus *Ectothiorhodospira* Pelsh 1936 to a New Family, *Ectothiorhodospiraceae* fam. nov., and Emended Description of the *Chromatiaceae* Bavendamm 1924. Int J Syst Bacteriol 1984; 34:338-339 10.1099/00207713-34-3-338

[32] Validation of the publication of new names and new combinations previously effectively published outside the IJSB. List No. 62. Int J Syst Bacteriol 1997; 47:915-916 10.1099/00207713-47-3-915 9336942

[33] Imhoff JF Süling J The phylogenetic relationship among Ectothiorhodospiraceae: a reevaluation of their taxonomy on the basis of 16S rDNA analyses. Arch Microbiol 1996; 165:106-113 10.1007/s002030050304 8593098

[34] Hirschler-Réa A Matheron R Riffaud C Mouné S Eatock C Herbert RA Willison JC Caumette P Isolation and characterization of spirilloid purple phototrophic bacteria forming red layers in microbial mats of Mediterranean salterns: description of *Halorhodospira neutriphila* sp. nov. and emendation of the genus *Halorhodospira.* Int J Syst Evol Microbiol 2003; 53:153-163 10.1099/ijs.0.02226-0 12656167

[35] Raymond JC Sistrom WR *Ectothiorhodospira halophila*: a new species of the genus *Ectothiorhodospira.* Arch Mikrobiol 1969; 69:121-126 10.1007/BF00409756 4192367

[36] Ashburner M Ball CA Blake JA Botstein D Butler H Cherry JM Davis AP Dolinski K Dwight SS Eppig JT Gene ontology: tool for the unification of biology. The Gene Ontology Consortium. Nat Genet 2000; 25:25-29 10.1038/75556 10802651 PMC3037419

[37] Edgar RC MUSCLE: multiple sequence alignment with high accuracy and high throughput. Nucleic Acids Res 2004; 32:1792-1797 10.1093/nar/gkh340 15034147 PMC390337

[38] Guindon S Gascuel O A simple, fast, and accurate algorithm to estimate large phylogenies by maximum likelihood. Syst Biol 2003; 52:696-704 10.1080/10635150390235520 14530136

[39] Chevenet F Brun C Bañuls AL Jacq B Christen R TreeDyn: towards dynamic graphics and annotations for analyses of trees. BMC Bioinformatics 2006; 7:439 10.1186/1471-2105-7-439 17032440 PMC1615880

[40] Dereeper A, Guignon V, Blanc G, Audic S, Buffet S, Chevenet F, Dufayard JF, Guindon S, Lefort V, Lescot M and others. Phylogeny.fr: robust phylogenetic analysis for the non-specialist. Nucleic Acids Res. 2008;36(Web Server issue):W465-9. PMID:18424797 10.1093/nar/gkn180 PMC2447785 18424797

[41] Markowitz VM Chen IM Palaniappan K Chu K Szeto E Grechkin Y Ratner A Jacob B Huang J Williams P IMG: the Integrated Microbial Genomes database and comparative analysis system. Nucleic Acids Res 2012; 40(Database issue):D115-D122 10.1093/nar/gkr1044 22194640 PMC3245086

